# Macrophage Perspectives in Liver Diseases: Programmed Death, Related Biomarkers, and Targeted Therapy

**DOI:** 10.3390/biom14060700

**Published:** 2024-06-14

**Authors:** Zibing Qian, Wanyuan Xiong, Xiaorong Mao, Junfeng Li

**Affiliations:** 1 The First Clinical Medical College of Lanzhou University, Lanzhou 730000, China; qianzb21@lzu.edu.cn (Z.Q.); xiongwy21@lzu.edu.cn (W.X.); 2 Department of Infectious Disease, The First Hospital of Lanzhou University, Lanzhou 730000, China; 3 Institute of Infectious Diseases, The First Hospital of Lanzhou University, Lanzhou 730000, China; 4 Department of Hepatology, The First Hospital of Lanzhou University, Lanzhou 730000, China

**Keywords:** liver diseases, macrophage, programmed cell death, biomarkers, targeted therapy

## Abstract

Macrophages, as important immune cells of the organism, are involved in maintaining intrahepatic microenvironmental homeostasis and can undergo rapid phenotypic changes in the injured or recovering liver. In recent years, the crucial role of macrophage-programmed cell death in the development and regression of liver diseases has become a research hotspot. Moreover, macrophage-targeted therapeutic strategies are emerging in both preclinical and clinical studies. Given the macrophages’ vital role in complex organismal environments, there is tremendous academic interest in developing novel therapeutic strategies that target these cells. This review provides an overview of the characteristics and interactions between macrophage polarization, programmed cell death, related biomarkers, and macrophage-targeted therapies. It aims to deepen the understanding of macrophage immunomodulation and molecular mechanisms and to provide a basis for the treatment of macrophage-associated liver diseases.

## 1. Introduction

According to the latest statistics, approximately 2 million people die from liver diseases annually, accounting for 3.5% of all deaths globally [1]. Both figures are unfortunately still on the rise. Across a wide range of liver diseases, including viral, metabolic, or autoimmune hepatitis, drug-induced hepatitis, and hepatocellular carcinoma, long-term, slow cellular changes lead to alterations in liver structure and function, along with the recruitment, disorganization, and death of immune cells. As crucial components of the body’s natural immune system, macrophages play an indispensable role in hepatic immune defense, tissue remodeling, and maintaining cellular dynamic homeostasis. Hepatic macrophages constitute the largest population of innate immune cells within the liver. In healthy rodent livers, these macrophages comprise approximately 20–25% of non-parenchymal cells and play an indispensable role in hepatic immune defense, tissue remodeling, and the maintenance of dynamic cellular homeostasis [2].

**Figure 1:**
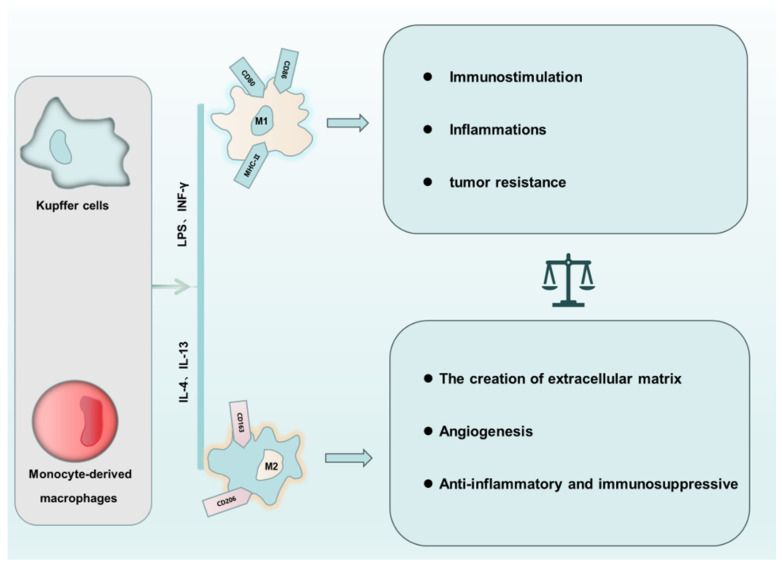
Phenotype and function of macrophages. Macrophages can be polarized by different factors into two distinct phenotypes (M1 and M2). Overall, M1 exhibits pro-inflammatory properties and M2 exhibits anti-inflammatory properties. The balance between the two determines the direction of liver Inflammation. IFN-γ: Interferon-γ; IL-4: Interleukin-4; IL-13: Interleukin-13; LPS: Lipopolysaccharide; MHC-II: Histocompatibility complex class II.

Hepatic macrophages can be categorized into two main populations based on their origin: Kupffer cells (KCs) and monocyte-derived macrophages (MoMFs) [3]. KCs originate primarily from embryonic yolk sac cells and bone marrow hematopoietic stem cells. These self-maintaining, locally proliferative cells contribute to immune tolerance. MoMFs, on the other hand, differentiate from circulating monocytes in the peripheral blood and are more responsive to signals that promote their functional specialization and infiltration into tissues. KCs and MoMFs play distinct roles in liver injury. KCs act as sentinels, phagocytosing harmful substances and regulating the immune response. MoMFs primarily produce inflammatory cytokines, thereby influencing both inflammation and trauma repair within the liver [4]. The cell surface of KCs expresses a diverse array of specific protein receptors, including mannose receptors (MR), scavenger receptors (SR), toll-like receptors (TLRs), nucleotide-binding oligomerization domain (NOD)-like receptors, and retinoic acid-inducible gene I (RIG-I)-like receptors. These receptors collectively equip KCs to recognize and eliminate invading pathogens, such as complement fragments detected by complement receptors (CRs) [5,6]. MoMFs are primarily recruited into the liver by the chemokine C-C motif ligand 2 (CCL2), also known as monocyte chemotactic protein 1. These macrophages are further classified into two subtypes based on their surface marker. Ly-6C: Ly-6C^high^ (Ly-6C^+^) MoMFs contribute to organ damage, while Ly-6C^low^ (Ly-6C^−^) MoMFs promote tissue repair [7]. During immune responses or pathological processes, macrophages alter their cellular phenotype and function in response to the specific microenvironment. These cells within an organism often coexist in varying ratios of M1-type and M2-type phenotypes, a process known as macrophage polarization [8] (Figure 1). M1 macrophages, primarily induced by Interferon-γ (IFN-γ), lipopolysaccharides (LPS), and Toll-like receptors (TLRs), secrete pro-inflammatory factors, participate in inflammatory responses, and exhibit pro-inflammatory, pathogen-clearing, and anti-tumor effects. In contrast, M2 macrophages, induced by Interleukin-4 (IL-4) and IL-13, release anti-inflammatory cytokines such as IL-10, transforming growth factor-β (TGF-β), and Arginase-1(Arg-1), playing roles in anti-inflammation, tissue remodeling promotion, and even accelerating tumor formation. Importantly, macrophage polarization is not static; these cells can dynamically switch phenotypes in specific environments [9]. M1 macrophages, characterized by their production of pro-inflammatory reactive oxygen species (ROS) and reactive nitrogen species, exacerbate organ damage in inflamed tissues. This destructive activity stems from the activation of NADPH oxidase 2 (NOX2) and inducible nitric oxide synthase (iNOS). Interestingly, these very weapons turn against M1 macrophages themselves, causing them to succumb to the ROS/reactive nitrogen species-rich environment they created, even causing macrophage death [10].

Cell death is a fundamental biological process that governs the development and regression of nearly all liver diseases and significantly impacts the severity and outcome of liver injury. Programmed cell death (PCD) pathways, such as apoptosis, necroptosis, autophagy, pyroptosis, and ferroptosis, exhibit distinct characteristics while also demonstrating numerous similarities and overlapping functions (Table 1). Macrophages, as the body’s major immune cells, play a regulatory role in phenotypic transformation through PCD. However, the relationship between PCD and macrophage polarization remains open to further investigation, with one potentially influencing the other. Considering the important impact of macrophages on hepatic inflammation and their role in the pathological progression of liver diseases, the programmed cell death (PCD) of macrophages must be closely related to the development of liver diseases. This review focuses on the effect of PCD on macrophage phenotype in liver diseases and its interrelationships, as well as the possibility of macrophage PCD for the treatment of liver diseases.

## 2. Macrophage Programmed Death

### 2.1. Macrophage Autophagy

Autophagy plays a crucial role in macrophage health by maintaining intracellular metabolic homeostasis. Under stress factors such as starvation, hypoxia, and infection, macrophages rapidly switch into “vacuum cleaner” mode, effectively removing metabolic waste products to restore cellular homeostasis [11]. Autophagy and its intricate link to inflammation and immunity have gained significant momentum in recent years. It is widely thought that enhanced autophagic activity may exert a protective effect by modulating the polarization phenotype of macrophages, thereby reducing the activation of inflammatory vesicles and the release of inflammatory factors. Additionally, it may influence macrophage apoptosis through mechanisms that are yet to be fully elucidated. Current research suggests a potential role for various signaling pathways, including nuclear factor kappa-B (NF-κB), rapamycin-targeted protein (mTOR), and class III phosphoinositide 3-kinase (PI3K)/protein kinase B (Akt). Within the autophagy process, the light chain 3 protein (LC3), formally known as microtubule-associated proteins light chain 3 (MAP1LC3), plays a crucial role. During autophagy, the LC3 protein is synthesized. Immediately after synthesis, the carboxyl terminus of the LC3 protein is sheared by Atg4, generating cytoplasmically localized LC3-I. LC3-I is modified and processed by a ubiquitin-like system that includes Atg7 and Atg3, generating LC3-II with a molecular weight of 14 kD that is localized to autophagosomes. Therefore, the presence of both LC3 and LC3-II in autophagosomes is regarded as a molecular marker of autophagy, and the amount of LC3-II is proportional to the degree of autophagy. Consequently, the LC3-II/LC3-I ratio serves as an indicator of autophagic activity [12,13]. Beclin-1, the mammalian orthologue of yeast Atg6, acts as a major positive regulator of autophagy. Under starvation conditions, JNK activation leads to the phosphorylation of Bcl-2, which in turn releases Beclin-1, thereby initiating autophagy [14]. The mTOR kinase family plays a critical regulatory role in the autophagic response. Activation of mTOR kinases leads to its inhibition, while conversely, inhibition of mTOR triggers the initiation of autophagy. The PI3K/AKT signaling pathway is one of the two upstream pathways regulating mTOR. Importantly, inhibition of PI3K can significantly block the downstream signaling mediated by AKT and mTOR [15]. Excessive accumulation of ROS disrupts cellular homeostasis, leading to both oxidative stress and mitochondrial dysfunction. Interestingly, this phenomenon can also induce autophagy [15]. Excessive accumulation of ROS disrupts cellular homeostasis, leading to oxidative stress and mitochondrial dysfunction, and autophagy is also induced [15]. Liu et al. [16] demonstrated that BML-111, a lipoxin A receptor agonist, stimulates autophagy in human alveolar macrophages by targeting mitogen-activated protein kinase (MAPK) signaling. This resulted in attenuated endotoxin LPS-induced apoptosis and facilitated the regression of acute lung injury. Furthermore, introducing free radicals (ROS or Nitric Oxide (NO)) into macrophages can lead to DNA strand breaks, ultimately triggering the AMP-activated Protein Kinase (AMPK)-Mechanistic target of rapamycin (mTOR) pathway to regulate autophagy. This process is also influenced by the overactivation of poly ADP-ribose polymerase, which depletes essential high-energy substrates such as NAD^+^ and ATP [17]. TcdB, a key exotoxin produced by Clostridioides difficile, activates macrophages, thereby promoting inflammation and epithelial damage. Vitamin D3 and carbamazepine have been demonstrated to attenuate TcdB-induced lysosomal dysfunction, inflammation, and histological damage. In a murine model of *Clostridioides difficile* infection (CDI), TcdB was found to inhibit the CTNNB1–MITF signaling axis, leading to suppressed lysosomal function. Conversely, it activated SQSTM1-NFκB signaling downstream of macrophages. Notably, the restoration of lysosomal function through these mechanisms contributed to the prevention of CDI in the mice [18]. Indeed, the current body of research data is primarily derived from animal models and in vitro experiments. The translatability of these findings to the human context remains unclear, and further investigations are warranted.

### 2.2. Macrophage Apoptosis

Apoptosis, the most common mode of macrophage death, serves as a crucial host defense mechanism. Factors such as foreign bacterial infection, viral infection, mycoplasma infestation, and changes in extracellular signaling molecules can reportedly trigger macrophage apoptosis [19]. One example involves macrophages infected with Mycobacterium tuberculosis. This infection leads to phosphorylation of the apoptosis inhibitory proteins FLIPs (FLICE/caspase8 inhibitory proteins), ultimately activating caspase-3/7 and triggering apoptosis [20]. Bacterial cell wall components can also induce macrophage apoptosis. For instance, the 19 kDa Mycobacterium tuberculosis lipoprotein can induce apoptosis in THP-1 cells through a TLR-dependent pathway [21,22]. Similarly, the Mycobacterium tuberculosis 38 kDa protein increases TNF-α levels, leading to the apoptosis of macrophages via the apoptosis-related factor ligand and caspase enzyme-dependent pathways [23]. Liver X receptors (LXRα and LXRβ), nuclear receptors controlling cholesterol metabolism and regulating macrophage differentiation, exhibit complex effects on apoptosis. While LXR activation enhances proinflammatory effects and impairs anti-inflammatory properties in macrophage colony-stimulating factor-dependent MoMFs [24], an abdominal macrophage study suggests that LXRα inhibits macrophage apoptosis by suppressing the endoplasmic reticulum stress-induced C/EBP homologous protein pathway [25]. The precise regulation of eukaryotic apoptosis during pathogen infection frequently constitutes a critical determinant in establishing a successful host–pathogen interaction [26]. Certain pathogens exploit this process by inducing apoptosis in macrophages, thereby evading their phagocytic activity. Conversely, apoptotic macrophages themselves can contribute to the inflammatory response by releasing pro-inflammatory cytokines, creating a complex feedback loop in this regulatory process.

### 2.3. Macrophage Necroptosis

Necroptosis, distinct from apoptosis and conventional necrosis, is a programmed cell death mode initiated by either tumor necrosis factor receptors (TNFRs) or pattern recognition receptors (PRRs). Two key proteins in this process are receptor-interacting protein (RIP) 1 and 3. Initiating necroptosis requires a series of molecules for death signal delivery and execution, including poly ADP-ribose polymerase-1, ROS, and Ca^2+^. These molecules ultimately lead to cellular necroptosis by damaging mitochondria and other organelles [27]. Unlike apoptosis, necroptosis exposes damage-associated molecular patterns (DAMPs) to the extracellular space, allowing phagocytes to recognize and clear them [27]. This process is facilitated by the mixed lineage kinase domain-like protein (MLKL), the most important downstream effector of necroptosis identified so far [28]. Phosphorylated MLKL converts from a monomeric to an oligomeric state, which binds phosphatidylinositol and myocardial phospholipids, resulting in the transfer of the entire binding to specific lipids. This complex then translocates from the cytoplasm to the cytosol or organelle membrane, where it forms permeable pores, ultimately rupturing the cell membrane [28]. Necroptosis releases cellular contents such as DNA fragments, ATP, and pro-inflammatory factors. These are recognized by PRRs, which activate the immune response in surrounding cells, eliminating the dead cells. Notably, a large amount of cell content spills after necroptosis, leading to inflammation. This response is prevented by the absence of RIP3 or MLKL [28]. Ni et al. [29] found that Concanavalin A (ConA)-induced macrophage death primarily occurs through necroptosis, not apoptosis.

### 2.4. Macrophage Pyroptosis

Macrophage pyroptosis, intricately linked to inflammatory factors such as IL-1β and IL-18, influences the intrinsic immune system and its role in diverse immune-related diseases is well-established [30]. Microbial structures and biological factors trigger caspase-1-mediated macrophage pyroptosis [31]. When encountering risk factors, macrophages experience oxidative stress, leading to gasdermin D (GSDMD) oxidation and NOD-like receptor family pyrin domain containing 3 (NLRP3) inflammasome activation. This reduces the mitochondrial membrane potential and generates ROS, with GSDMD oxidation promoting NLRP3-dependent pyroptosis via mitochondrial reactive oxygen species (mtROS) [31]. Studies exploring macrophage death mechanisms have revealed diverse triggers and pathways. Stable transfection of THP-1 cells with Siglec-14, for instance, leads to caspase-1 activation, NLRP3 inflammasome activation, and IL-1β release, ultimately causing cellular pyroptosis upon group B streptococcal infection [32]. Similarly, isoproterenol-induced pyroptosis activates macrophages through the NLRP3/ASC/caspase-1 pathway, suggesting a potential target for mitigating its adverse effects during use and offering new insights into anesthetic drug applications [33]. Conversely, quercetin demonstrates a protective effect against THP-1 macrophage pyroptosis by decreasing NLRP3, cleaved-caspase1, IL-1β, and N-GSDMD expression in a dose-dependent manner. Interestingly, AMPK agonists counteract this beneficial effect by weakening quercetin’s inhibition of NLRP3 inflammasome activity [34]. Finally, LPS co-treatment with Nigerian bacteriocin has been shown to induce caspase-1-dependent pyroptosis in THP-1 cells via the formation of NLRP3 inflammasome vesicles [35].

### 2.5. Macrophage Ferroptosis

The synergistic interaction between iron and other hepatotoxins (e.g., alcohol, drugs, viruses, lipids) exacerbates the liver’s vulnerability to damage. Even slightly elevated tissue iron levels can potentiate the harmful effects of these insults, accelerating liver disease progression [36]. Lipid peroxidation plays a critical role in the function and survival of various immune cells, participating in both their execution through ferroptosis and the pathology of various diseases. Excess lipid peroxide accumulation in immune cells can trigger their own ferroptosis, directly impacting their function. Additionally, non-immune cells can induce ferroptosis in immune cells through lipid peroxidation and the release of signaling molecules, with both processes influencing organismal homeostasis. Youssef et al. [37] demonstrated that macrophages undergo lipid peroxidation and increased ROS, ultimately leading to ferroptosis following enhanced erythrocyte phagocytosis. Exposure to cadmium telluride quantum dots triggered cell death in RAW264.7 cells via activated ferritinophagy. This involved nuclear factor erythroid 2-related factor 2 (NRF2) downregulation, extracellular signal-regulated kinases (ERK1/2) phosphorylation, and subsequent ferritin heavy chain 1 degradation in lysosomes, ultimately causing the release of free iron ions and initiating macrophage ferroptosis [38].

## 3. The “Love of Kill” between Macrophage Polarization and PCD

The complex interplay between macrophage polarization and programmed cell death involves a dynamic power struggle. The PCD of macrophages plays a critical role in regulating their phenotypic transformation. Furthermore, a distinct connection exists between different PCD pathways. Macrophages exhibit plasticity, polarizing into M1 and M2 phenotypes in response to specific stimuli. LPS and IFN-γ activate M1 macrophages, promoting inflammatory responses. Conversely, IL-4 and IL-13 induce M2 polarization, which promotes anti-inflammatory processes and tissue repair. The susceptibility of macrophages to various forms of PCD can be influenced by their polarization status, the level of microenvironmental inflammation, and the presence of ROS and peroxides [39,40]. While macrophage autophagy often promotes M1 polarization, leading to chronic inflammation and liver damage in obese mice, the vulnerability of these M1 macrophages to specific PCD pathways varies [41]. Bone marrow monocyte-derived macrophages (BMDMs) of the M1 phenotype were found to be more susceptible to necroptosis compared to the M0 and M2 subtypes, as indicated by the presence of necroptosis-dependent receptor-interacting protein kinase-3 (RIPK3) activity in M1 macrophages [42]. In contrast, Kapralov et al. [43] demonstrated that M1 macrophages exhibited greater resistance to ferroptosis compared to their M0 and M2 counterparts, which was attributed to several factors in M1 macrophages: high levels of inducible iNOS, increased NO production, inhibition of 15-lipoxygenase, and membrane diffusion of NO. Interestingly, this NO diffusion also conferred ferroptosis resistance to surrounding cells near M1 macrophages, mimicking the protective effect of Glutathione peroxidase 4 (GPX4). DMF, a pyroptosis inhibitor, not only alleviated acute kidney injury in crush syndrome but also suppressed M1 macrophage polarization through the Caspase1/GSDMD signaling pathway [44]. Another study revealed that M2-type KCs could promote the apoptosis of M1-type KCs through an IL-10-mediated, arginase-dependent mechanism [45]. This finding highlights the intricate interplay between macrophage polarization and programmed cell death pathways. Considering such interactions, future research could explore the potential of modulating specific PCD pathways in different macrophage subtypes as a therapeutic strategy for immunoinflammatory diseases. This might involve either enhancing the programmed cell death resistance of specific macrophage types in certain diseases, such as inducing M2-type macrophage death in hepatocellular carcinoma, or promoting death in detrimental macrophage populations, such as inducing M1-type macrophage death in non-tumorigenic diseases.

## 4. Interactions between Different Types of PCD in Macrophages

**Figure 2:**
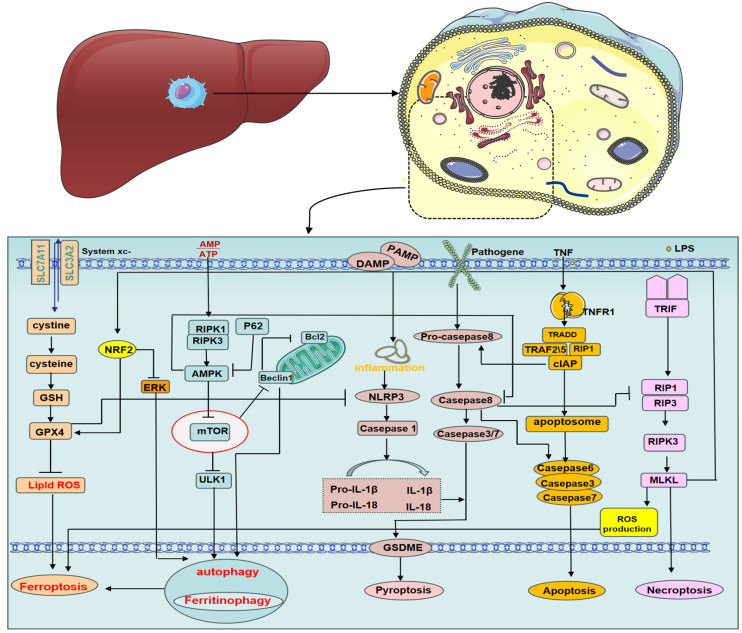
Association of different types of PCD in macrophages. The interactions between the different PCD types of macrophages constitute a complex regulatory network for survival and/or death. SLC3A2: Solute carrier family 3 member 2; SLC7A11: Solute carrier family 7 member 11; GPX4: Glutathione peroxidase 4; GSH: Glutathione; ROS: Reactive oxygen species; NRF2: Nuclear factor erythroid 2-related factor 2; ERK: Extracellular signal-regulated kinases; PIPK1: Phosphatidylinositol phosphate kinase 1; PIPK3: Phosphatidylinositol phosphate kinase 3; AMPK: AMP-activated Protein Kinase; mTOR: Mechanistic target of rapamycin; ULK1: UNC-51-like kinase 1; BCL2: B-cell lymphoma 2; MLKL: Mixed lineage kinase domain-like protein; DAMPs: Damage-associated molecular patterns; PAMP: Pathogen-associated molecular pattern; GSDME: Gasdermin E; NLRP3: NOD-like receptor family pyrin domain containing 3; TNF: Tumor necrosis factor; TNFR1: Tumor Necrosis Factor Receptor-1; TRADD: TNFR1-associated death domain protein; TRAF2: TNF receptor associated factor 2; TRAF5: TNF receptor associated factor 2; RIP1: Receptor-interacting protein 1; TRIF: Toll/interleukin 1 receptor-domain-containing adapter-inducing interferon-β; RIP1: Receptor-interacting protein kinase 1; RIP3: Receptor-interacting protein kinase 1.

In multicellular organisms, the balance between cell survival, proliferation, and death is strictly regulated. Macrophage PCD plays a critical role in their sentinel and innate immune functions, and there is significant crosstalk between different types of macrophage PCD (Figure 2). In some cases, PCD facilitates intracellular pathogen clearance and alerts the immune system to resist invasion [10]. However, macrophage death can also lead to the release of danger signals, both directly and indirectly, through other dead cells [46]. Necroptosis, a key regulator of macrophage function and death, serves as an alternative form of cell death when apoptosis fails [47]. Activated MLKL during necroptosis triggers NLRP3 inflammasome assembly within the cell, while RIPK3 can also activate inflammasomes independently of MLKL [48,49,50]. Similarly, MLKL knockdown leads to aberrant RIP3 accumulation and consequent caspase-8 activation, causing cell death [51]. The relationship between autophagy and necroptosis is complex and not solely antagonistic. RIPK3 can directly bind and activate AMPK to promote autophagy [52], and the MLKL inhibitor necrosulfonamide can also inhibit pyroptosis [53]. Upstream of RIPK3, RIPK1 regulates apoptosis and necroptosis through caspase-8 and RIPK3, respectively [54], while caspase-8 itself can induce pyroptosis by cleaving GSDMD [55]. These studies highlight the intricate network of interactions between cellular pyroptosis, apoptosis, and necroptosis. The relationship between autophagy and ferroptosis is well-established. mTOR, an autophagy inhibitor, also inhibits ferroptosis by regulating GPX4 synthesis [56], while liensinine protects against acute lung injury by enhancing M2 macrophage resistance to ferroptosis through autophagy regulation [57]. Interestingly, both pyroptosis and autophagy promote ferroptosis, but not each other, exhibiting an antagonistic relationship. Tim-4-mediated macrophage autophagy directly inhibits macrophage pyroptosis and indirectly suppresses it by modulating CD8+ T cell activation [58]. Additionally, a ferroptosis inhibitor (Fer-1) can block various inflammatory responses by inhibiting cytokine production [59]. Notably, GPX4 acts as a defect-dependent regulator of NLRP3-mediated pyroptosis [60]. Most cellular ROS production occurs in the mitochondria. Activated ROS promote the formation of ceramide-rich lipid rafts and death receptors, leading to caspase-8 activation and apoptosis. ROS also activate histone D, caspase-8, and caspase-3, further triggering apoptosis. Furthermore, ROS play a role in various PCDs, including pyroptosis, autophagy, necroptosis, and ferroptosis [61].

## 5. Effect of Macrophage PCD on Liver Disease

**Figure 3:**
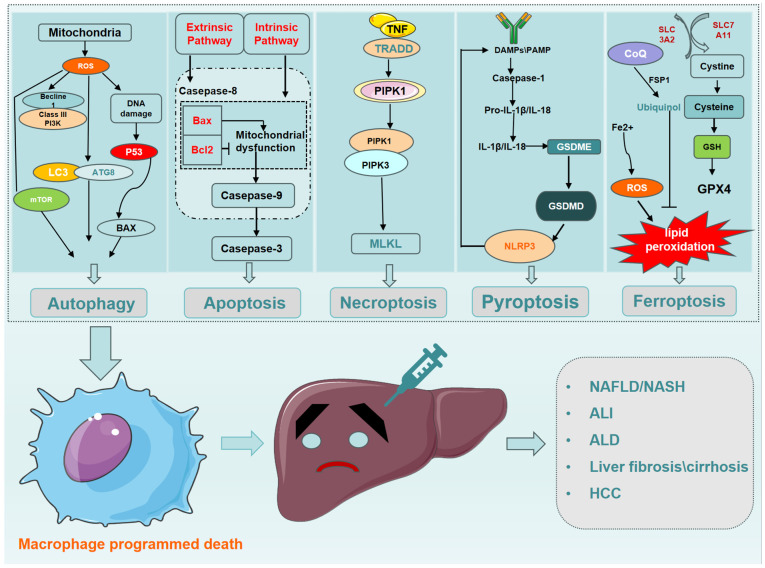
Macrophage PCD is involved in the regulation of liver disease. Macrophages are induced by different stimuli or signals to produce different forms of death, including autophagy, apoptosis, necroptosis, pyroptosis, and ferroptosis, and thus play a regulatory role in the occurrence and development of diseases. ROS: Reactive oxygen species; Class III PI3K: Class III phosphoinositide 3-kinase; mTOR: Mechanistic target of rapamycin; LC3: Microtubule-associated protein light chain 3; ATG5: Autophagy protein 5; Bcl-2: B-cell lymphoma 2; TNF: Tumor necrosis factor; TRADD: TNFR1-associated death domain protein; PIPK1: Phosphatidylinositol phosphate kinase 1; PIPK3: Phosphatidylinositol phosphate kinase 3; MLKL: Mixed lineage kinase domain-like protein; DAMPs: Damage-associated molecular patterns; PAMP: Pathogen-associated molecular pattern; NLRP3:NOD-like receptor family, pyrin domain containing 3; GSDME: Gasdermin E;SLC3A2: Solute carrier family 3 member 2; SLC7A11: Solute carrier family 7 member 11; CoQ: Coenzyme Q; FSP1: Ferroptosis suppressor protein 1; GPX4: Glutathione peroxidase 4.

Cell death plays a key role in driving the progression of various forms of liver disease. The association between PCD and different types of liver disease is expected to provide a new therapeutic strategy for the diagnosis, treatment, and prognosis of liver diseases. Here, we will discuss the mechanisms of macrophage PCD and its regulation in liver disease (Figure 3).

### 5.1. The Effect of Macrophage PCD on NAFLD/NASH

A staggering 22–28% of the global population suffers from non-alcoholic fatty liver disease (NAFLD), with its progression to cirrhosis and hepatocellular carcinoma (HCC) becoming a major driver of clinical liver transplantation [62]. Despite burgeoning research on NAFLD/non-alcoholic steatohepatitis (NASH) in recent years, no approved drug therapies exist. Therefore, a deep understanding of the disease’s cellular mechanisms is crucial to developing novel therapeutic strategies and alleviating the growing global healthcare burden.

#### 5.1.1. Autophagy

Excessive lipid accumulation disrupts cellular autophagy, consequently modulating the immune response. Conversely, autophagy can also influence innate immune activation [41]. Studies have shown that impaired macrophage autophagy in mice fed a high-fat diet promotes inflammatory responses, which can be sufficient to trigger liver injury [41]. Moreover, research using a methionine- and choline-deficient L-amino acid diet (MCD) model and clinical NASH patients revealed complex interactions between HIF-1α and autophagy in macrophage activation. Both pathways contribute to the proinflammatory hyperactivation observed in NASH due to the MCD diet [63]. While HIF-1α and AMPK/mTORC1 are known to regulate macrophage autophagy and phenotypic transformation, respectively, their interplay in the context of hypoxia and energy regulation remains poorly understood. Further investigation is needed to fully elucidate these interactions. Additionally, macrophage autophagy suppresses the expression of programmed cell death ligand 1 (PD-L1), thereby hindering hepatocellular carcinoma progression [64]. Considering the increased incidence of NAFLD-associated HCC, modulation of macrophage autophagy may be valuable in reducing NAFLD-associated HCC.

#### 5.1.2. Apoptosis

Alpha-1-microglobulin (AIM) plays a critical role in maintaining tissue homeostasis and regulating inflammation. Primarily produced and secreted by macrophages, with additional release from the bloodstream, AIM exerts its intracellular effects through mechanisms such as lipid metabolism and apoptosis. Interestingly, AIM’s influence extends beyond its established roles in autoimmune diseases, hepatocyte targeting in hepatocellular carcinoma, and dead cell clearance in various conditions. Studies have linked AIM to pulmonary diseases, sepsis, atherosclerosis, cardiovascular events, and even exacerbated inflammatory responses [65]. It is well-established that AIM exhibits diverse in vivo functionalities, but the specific mechanisms underlying its beneficial or detrimental effects on inflammation remain elusive. M2 macrophages typically counteract the pro-inflammatory functions of M1 macrophages to suppress inflammation. This process likely involves inhibiting pro-inflammatory signaling pathways. In a high-fat diet-induced NAFLD mouse model, IL-4-activated M2-type KCs released IL-10 and induced apoptosis in M1-type KCs, thereby reducing liver inflammation and hepatocyte injury [45]. This finding suggests an intrinsic regulatory mechanism where M2 macrophages promote protective apoptosis in M1 macrophages, creating a link between KC polarization and fatty liver disease prevention. Additionally, this study raises the intriguing possibility that individual susceptibility to NAFLD and other conditions might be influenced by variations in KC phenotype alongside environmental, genetic, and metabolic factors.

#### 5.1.3. Necroptosis

Despite limited research on macrophage necroptosis in liver disease, several high-quality studies have emerged. Receptor-interacting protein 1 (RIP1), a crucial player in apoptosis, necroptosis, and inflammation, stands out as a potential therapeutic target [27]. Feeding high-fat diets to wild-type and RIP1 kinase-dead (Rip1 K45A/K45A) mice revealed a striking difference: Rip1 K45A/K45A mice exhibited significantly reduced liver steatosis, injury, and fibrosis, accompanied by lower levels of hepatocellular cell death and inflammation compared to their wild-type counterparts. Both in vivo and in vitro studies demonstrated that lipotoxicity and saturated fatty acid (palmitic acid) treatment activated RIP1 kinase in hepatic macrophages. Furthermore, RIP1 kinase mediation was crucial for inflammasome activation, apoptosis, and necroptosis induced by palmitate in BMDMs and KCs. Adding clinical relevance, RIP1 kinase was significantly activated in the livers of patients with NASH, and this activation primarily occurred in hepatic macrophages [66]. This compelling evidence provides direct genetic proof that macrophage RIP1 kinase is a specific and potentially targetable contributor to NASH progression.

#### 5.1.4. Pyroptosis

While most research on pyroptosis in liver diseases focuses on hepatocytes, inflammatory bodies are predominantly expressed in immune cells, particularly macrophages. Studies have shown that knocking down NRF2 specifically in macrophages induces macrophage pyroptosis and worsens NASH progression by boosting ROS and IL-1β production through a YAP-NLRP3-dependent pathway [67]. Similarly, another study demonstrated that caspase-11 deficiency reduces BMDM pyroptosis and disrupts both glycolysis and oxidative phosphorylation in macrophages stimulated by palmitic acid [68]. This suggests that caspase-11 plays a key role in maintaining these metabolic processes to fuel macrophage pyroptosis. These findings not only highlight the significance of the caspase-11/GSDMD pathway in driving inflammation and pyroptosis in hepatic macrophages but also offer novel therapeutic targets for various stages of liver disease progression, providing new targets for the transition from NAFLD to NASH and subsequent progression to cirrhosis and hepatocellular carcinoma, as well as developing future therapeutic interventions for liver transplantation.

#### 5.1.5. Ferroptosis

Approximately one-third of patients with non-alcoholic fatty liver disease and metabolic syndrome test positive for dysmetabolic iron overload syndrome, a marker of iron overload [69]. Clinical studies also demonstrate a correlation between iron deposition in macrophages and the severity of NASH and advanced histological features of the liver [70]. This suggests that ferroptosis, a form of cell death triggered by iron overload, may contribute to NAFLD/NASH progression. A study employing magnetic column separation identified iron-rich KCs exhibiting pro-inflammatory and fibrotic phenotypes during NASH development [71]. The researchers also discovered a unique subpopulation of iron-rich macrophages contributing to the “corona”, an intrahepatic structure formed by macrophages surrounding dead hepatocytes. These macrophages clear debris, trigger inflammation and fibrosis, and are considered drivers of hepatic fibrosis in NASH. Interestingly, monounsaturated fatty acids, synthesized de novo within cells and incorporated into membrane lipids, act as ferroptosis inhibitors. Studies revealed the upregulation of sterol regulatory element-binding protein 1 in M1 macrophage expression, a key regulator of MUFA synthesis. This suggests that ferroptosis signaling might induce M1 polarization in macrophages, prompting metabolic reprogramming to enhance their ferroptosis resistance. Similarly, M2 macrophages exhibit increased fatty acid carboxylation and expression of lipid transporter proteins such as CD36 [72]. Carboxylation reduces polyunsaturated fatty acid accumulation and suppresses lipid peroxidation, while CD36-mediated lipid uptake increases susceptibility to ferroptosis [73]. Emerging evidence suggests that ferroptosis occurs during the early stages of NASH. Notably, the inhibition of ferroptosis has been demonstrated to offer near-complete protection against necrotic death in hepatocytes [74]. These findings substantiate that ferroptosis not only plays a role in NAFLD but also accelerates the progression of liver lesions. Mechanistically, ferroptosis is characterized by a massive accumulation of lipid peroxides within the cell membrane, starting at the inner membrane and progressing to the plasma membrane. This accumulation disrupts cellular homeostasis by triggering lethal ionic imbalances and increased membrane permeability. This evolving understanding of ferroptosis paves the way for novel therapeutic strategies that modulate lipid metabolism to either promote or inhibit ferroptosis. However, significant gaps remain in our knowledge regarding the precise mechanisms by which ferroptosis contributes to inflammation and how ferroptosis and lipid metabolism interact and transition within the context of NASH.

### 5.2. The Effect of Macrophage PCD on Alcoholic Liver Disease

The low awareness and diagnosis rate of alcoholic liver disease (ALD) often result in delayed diagnosis, with patients only recognized when they reach advanced stages such as cirrhosis or even liver failure. This highlights the importance of understanding ALD pathogenesis for timely diagnosis and effective treatment. While research on macrophage programmed cell death in ALD primarily focuses on autophagy and apoptosis, exploring other macrophage death modes remains a relatively untouched area [75].

#### 5.2.1. Autophagy

It is now understood that alcohol disrupts autophagic flow by affecting multiple genes involved in the autophagy process [76]. Interestingly, studies suggest that activating KCs through cannabinoid receptor 2 protects against alcohol-induced liver injury. This protective effect is mediated by an autophagy-dependent pathway involving heme oxygenase-1, which is known to dampen hepatic inflammatory responses [77]. However, further research is needed to fully understand the role of macrophage autophagy in ALD. Using a monocyte-macrophage autophagy-specific knockout model (Atg7f/f;Lyz-Cre mice), researchers found that these mice develop more severe liver injury after alcohol feeding compared to control mice. This increased injury was associated with mitochondrial ROS overproduction, elevated secretion of inflammatory cytokines (IL-1β, CCL5, and CXCL10), and steatosis. The study suggests that this damage arises primarily from dysregulation of intracellular interferon regulatory factor (IRF) levels due to impaired degradative autophagy, ultimately leading to dysregulated CCL5 and CXCL10 secretion and contributing to liver injury [78]. Prior research has primarily concentrated on the impact of impaired hepatocyte autophagy in the pathogenesis of alcoholic liver disease. While the activation of the innate immune response is recognized as a critical factor in the development of this disease, the role of macrophage autophagy in ALD remains less elucidated. An alternative therapeutic approach may lie in augmenting macrophage autophagy to dampen detrimental inflammatory responses [79]. This strategy, potentially offering the dual benefit of mitigating cellular damage and downregulating inflammation, represents a promising new direction for ALD treatment.

#### 5.2.2. Apoptosis

Adenosine receptor A2B (A2BAR), a negative regulator of inflammation, emerged as the most highly expressed adenosine receptor in the liver tissues of alcohol-fed mice. In vivo stimulation by injecting an A2BAR agonist increased cyclic adenosine monophosphate (cAMP) levels and attenuated alcohol-induced steatosis and inflammation. Conversely, knocking down A2BAR expression exacerbated the alcohol-induced inflammatory response in RAW264.7 cells. Overexpressing A2BAR significantly increased cAMP levels and impacted the expression of phosphorylated proteins involved in the NF-κB signaling pathway, particularly decreasing levels of the key protein p-P65. Notably, modulating A2BAR expression in alcohol-exposed RAW264.7 cells co-cultured with AML-12 hepatocytes led to a significant change in hepatocyte apoptosis rate, as measured by flow cytometry [80]. Studies have demonstrated that ethanol exposure promotes macrophage apoptosis in a time-dependent manner [81]. TGF-β, known to regulate various forms of apoptosis, may play a partial role in this process. It is now understood that ethanol can enhance macrophage TGF-β expression, as evidenced by the ability of anti-TGF-β antibodies to inhibit ethanol-induced macrophage apoptosis. Interestingly, patients with persistent alcohol consumption and steatotic liver injury exhibit elevated M2 macrophage gene expression, with negligible hepatocyte apoptosis but significant macrophage apoptosis [45]. This finding suggests a novel mechanism for the elimination of M1 macrophage populations, wherein M2 macrophages selectively induce M1 apoptosis. These combined research efforts have significantly advanced our understanding of the mechanisms underlying macrophage apoptosis in ALD. It is anticipated that such advancements will not only raise public awareness of the detrimental effects of alcohol but also foster a heightened level of vigilance regarding its consumption.

#### 5.2.3. Necroptosis

Macrophages are activated in response to a diverse array of stimuli, including pathogen-associated molecular patterns (PAMPs), DAMPs, toxins, cytokines, and chemokines. Notably, bacterial pathogens can exploit macrophages to subvert host defenses by inducing their necroptosis. For instance, excess TNF-α has been shown to increase RIPK1-RIPK3-dependent mitochondrial ROS production in Mycobacterium tuberculosis-infected macrophages. Here, cyclophilin D (CypD) plays a crucial role by regulating the formation of mitochondrial membrane pores and ceramide synthesis, ultimately triggering necroptosis [82]. Additionally, Mycobacterium tuberculosis secretes a virulence factor called tuberculosis necrotizing toxin (TNT), a nicotinamide adenine dinucleotide (NAD+) glycohydrolase that directly induces necroptosis in infected macrophages [27]. In the context of ALD, elevated plasma endotoxin levels have been documented in both human patients and animal models [83,84]. Endotoxin, a lipopolysaccharide component of the outer membrane in gram-negative bacteria, can promote intestinal permeability upon endotoxemia. This increased permeability allows endotoxin to translocate from the gut lumen into the bloodstream and ultimately reach the liver. Within the liver, endotoxin interacts with and activates hepatic macrophages, leading to the production of superoxide and TNF-α. These factors contribute to liver injury and potentially even cellular necroptosis [85]. However, the precise mechanisms underlying macrophage necroptosis in ALD remain complex and require further investigation. While anti-inflammatory macrophages can help mitigate alcohol-induced inflammation, their prolonged activation holds detrimental consequences. Such sustained activation can stimulate stellate cells, significantly contributing to liver fibrosis, a hallmark of ALD. Given their central role in the hepatic response to injury, any degenerative changes or death of macrophages can trigger a self-perpetuating cycle. These dying macrophages release inflammatory mediators that orchestrate further inflammation, highlighting the intricate interplay between cell death and inflammatory processes.

### 5.3. The Effect of Macrophage PCD on Acute Liver Injury

Acute liver injury (ALI), a complex condition triggered by diverse factors such as drug toxicity, viral infections, immune reactions, alcohol, and chemical toxins, poses a significant therapeutic challenge. Currently, no specific drugs or treatments can fully restore liver function in ALI patients, leaving liver transplantation as the only definitive option. However, this approach is hampered by limitations such as scarce donor organs, lifelong immunosuppressive therapy, high costs, and technical complexities. Therefore, the development of safe and potent drugs to improve ALI treatment and patient outcomes is of paramount importance.

#### 5.3.1. Autophagy

The LPS/D-GalN-induced acute severe hepatitis model is widely used to study the mechanisms of acute liver injury [86]. In this model, LysM-CRE-mediated macrophage autophagy ATG5 knockout mice displayed significantly increased serum alanine ALT, liver injury according to histological analysis, caspase activation, and mortality compared to non-knockout mice. Cultured hepatic macrophages from LPS/D-GalN-treated knockout mice also exhibited increased IL-1β production, suggesting that dysregulated IL-1β contributes to aggravated liver injury [87]. Another study examined the morphological changes of mouse peritoneal macrophages upon incubation with horseradish peroxidase-conA (conA-HRP). The experiment revealed that conA-HRP entered vesicles through receptor-mediated endocytosis, forming various endosome structures including vesicular, tubular, and double-membrane linear types. The double-membrane linear structures encapsulated part of the cytoplasm and organelles, marking them as autophagosomes. Fusion of these autophagosomes with lysosomes ultimately led to macrophage apoptosis [88]. The above studies highlight the necessity of further elucidating the role of macrophage autophagy in liver inflammation and injury. A deeper understanding of this process may unveil a promising therapeutic target for human liver disease. Indeed, most prior research has focused on how alterations in the autophagy of other hepatic parenchymal cells influence macrophage activation and hepatic inflammation. By shifting the research focus towards macrophage autophagy, a dual therapeutic benefit may be achievable.

#### 5.3.2. Necroptosis

While studies have demonstrated an increase in necroptosis during ConA-induced liver injury [89], the precise role of necroptosis in this model continues to be a subject of debate. ConA, a well-established mouse model, closely mimics human immune-mediated hepatitis in its pathogenesis, making it valuable for studying conditions such as autoimmune hepatitis and acute viral hepatitis [90]. This model exhibits unique features, including significantly elevated liver enzymes (alanine aminotransferase (ALT) and aspartate transaminase (AST)), infiltration of various immune cells (macrophages, neutrophils, T cells, and natural killer T cells), and increased inflammatory cytokines (TNF-α and IFN-γ). These factors contribute to extensive hepatic necroptosis, even affecting immune cells themselves [90]. Studies have specifically identified increased macrophage necroptosis in ConA-induced liver injury. Interestingly, z-Val-Ala-DL-Asp-fluoromethylketone (zVAD), a pan-caspase inhibitor, was found to induce macrophage necroptosis paradoxically. This seemingly counterintuitive effect was explained by zVAD-mediated upregulation of TNFR1 expression on macrophages through IL-10 signaling, ultimately increasing their susceptibility to necroptosis [29]. This research not only sheds new light on the mechanisms of zVAD-induced necroptosis but also offers promising avenues for treating acute liver injury. Future investigations aimed at elucidating the role of macrophage necroptosis within the hepatic inflammatory response have the potential to yield novel therapeutic strategies and deepen our understanding of the pathogenesis of various acute liver diseases.

#### 5.3.3. Pyroptosis

EuHD1, a newly developed nonsteroidal anti-inflammatory drug (NSAID), offers a promising alternative to traditional NSAIDs with its improved gastrointestinal safety profile. In vitro studies demonstrated that EuHD1 effectively inhibited macrophage pyroptosis induced by the combination of LPS and ATP. Mechanistically, it blocked the activation of NLRP3 inflammasomes, caspase-1, and subsequent IL-1β secretion. Additionally, EuHD1 alleviated LPS/GalN-induced ALI in mice, suppressing oxidative stress (SOD/MDA levels) and blocking NLRP3 inflammasome activation [91]. Interestingly, levels of pyroptosis-related proteins, particularly GSDMD-N, were significantly elevated in the liver tissues of ALI patients. Furthermore, GSDMD knockdown in LPS/GalN-induced ALI mice significantly reduced hepatic inflammation and improved survival rates. Unlike the typical immune cell pyroptosis that releases IL-1β and IL-18, GSDMD-mediated hepatocyte pyroptosis exacerbates cell death by recruiting macrophages via the MCP1/CCR2 pathway [92]. This highlights the involvement of macrophages in hepatocyte pyroptosis during acute liver injury. ALI is characterized by a well-established association with disruptions in innate immune function and acute hepatocellular damage. In this context, macrophage pyroptosis has emerged as a critical player in the pathogenesis of ALI [93]. Studies employing a mouse model of septic ALI induced by cecum ligation puncture (CLP) have revealed a significant proportion of cells undergoing pyroptosis, with 18.19% representing the total cell population and 16.29% specifically confined to hepatic macrophages [94]. These findings collectively highlight the pivotal role of macrophage pyroptosis across various etiologies of ALI, including sepsis and drug-induced injury. Consequently, targeting this specific cell death pathway in macrophages presents a promising therapeutic avenue for mitigating ALI.

#### 5.3.4. Ferroptosis

M2-type macrophages, known for their role in inflammation and recovery from ALI [57], may be susceptible to ferroptosis, potentially affecting survival and liver function in LPS/GalN-induced ALI. Investigating the potential therapeutic effects and mechanisms of action of liensinine in ALI, researchers observed an increased survival rate and reduced inflammatory factor production in LPS/GalN-treated mice treated with liensinine. Furthermore, liensinine significantly reduced ferroptosis inducer RSL3-induced lipid peroxidation in M2 macrophages, inhibited ferritinophagy, and blocked Fe^2+^ synthesis. Transmission electron microscopy revealed a close association between ferritin and microtubule-associated protein light chain 3 (LC3)-positive vesicles in M2 macrophages treated with both RSL3 and liensinine. Separately, Chen et al. demonstrated that administration of antibodies against histone H3 blocked macrophage ferroptosis activation in ALI mice, protecting them from severe liver injury. Histone H3, interacting with the cell membrane’s phospholipid bilayer, can induce cell death by compromising membrane barrier function [95]. Macrophages, a critical population of immune cells, orchestrate both inflammatory responses and iron homeostasis. Consequently, the precise regulation of macrophages represents a potential avenue for influencing the development of ferroptosis. Future research endeavors should prioritize the identification of specific targets within the ferroptosis pathway. Elucidating these targets holds the potential to unlock novel therapeutic strategies for the treatment of acute liver failure.

### 5.4. The Effect of Macrophage PCD on Liver Fibrosis/Cirrhosis

Liver fibrosis, a hallmark of chronic liver disease, often relentlessly progresses to cirrhosis, affecting outcomes. These outcomes depend on multiple factors, including the underlying disease, access to appropriate treatment, and the individual’s disease course. Notably, a European study revealed a high prevalence of undetected advanced fibrosis in asymptomatic individuals, suggesting that the true burden of liver fibrosis/cirrhosis might be significantly underestimated [96]. This alarming finding underscores the urgent need to better understand the disease mechanisms and develop effective therapeutic strategies.

#### 5.4.1. Autophagy

Mounting evidence suggests that macrophage autophagy acts as a negative regulator of inflammation. This suppressive effect is achieved by limiting the release of inflammatory factors from macrophages and hindering the recruitment of inflammatory cells. Macrophage infiltration and their cytokine production, particularly IL-1α and IL-1β, not only directly activate hepatic stellate cells (HSCs) but also exacerbate liver inflammation and fibrosis. Studies have shown that macrophage-specific deletion of autophagy protein 5 (ATG5) increases these pro-inflammatory cytokines and worsens liver damage in mice [97]. Furthermore, T-cell immunoglobulin and mucin domain-4 (TIM-4), expressed in macrophages and dendritic cells, appear to play a protective role. Regulating TIM-4 in KCs inhibits ROS production, thereby reducing LC3-II/I activation and TGF-β1 secretion, ultimately attenuating liver fibrosis [98]. Another crucial process linked to fibrosis is enhanced LC-3-associated phagocytosis (LAP) in hepatic mononuclear cells. Interestingly, inhibiting LAP in patients with liver fibrosis/cirrhosis worsens these conditions, highlighting its potential therapeutic significance [99]. Finally, the interplay between ROS, NRF2, and autophagy emerges as a critical pathway influencing macrophage response in liver fibrosis. ROS activates NRF2, which induces p62-dependent selective autophagy in macrophages [100]. However, autophagy-deficient macrophages promote liver damage by enhancing the mtROS/NF-κB/IL-1a/b pathway [101]. In addition, macrophage autophagy extends beyond the degradation of cellular components and encompasses a broader range of physiological mechanisms. These mechanisms include the cytosolic burial of damaged organelles, the prevention of oxidative stress, and the modulation of inflammatory cytokine production, particularly IL-1A and IL-1B [97,102]. Beyond its role in regulating liver diseases, macrophage autophagy has emerged as a promising therapeutic target for various inflammatory diseases such as colitis, atherosclerosis, and tuberculosis.

#### 5.4.2. Apoptosis

Hepatic macrophages contribute to the pathogenesis of liver fibrosis by secreting pro-inflammatory cytokines, such as TNFα [12,103]. Additionally, studies have demonstrated that hepatic macrophages in fibrotic livers undergo apoptosis, potentially through a Fas-mediated pathway. CHI3L1, largely found in hepatic macrophages, accumulates significantly in liver fibrosis, potentially contributing to increased serum CHI3L1 levels. A study demonstrated that CHI3L1 protects these macrophages from apoptosis by suppressing Fas, an apoptosis-related factor, and the Akt signaling pathway [104]. Interestingly, while AIM and adipocytokines are linked to metabolic syndrome and potentially influence chronic hepatitis C progression, only serum AIM levels above 1.2 μg/mL were independently associated with advanced fibrosis and not with steatosis or inflammation [105]. A study has shown that patients with mild liver injury exhibit a higher incidence of hepatic macrophage apoptosis compared to those with severe injury [45]. In addition, research suggests that hepatic macrophage apoptosis may be protective against the progression of steatohepatitis [106]. Interestingly, depletion of hepatic macrophages has been demonstrated to ameliorate hepatic fibrosis, particularly during the progressive inflammatory stage of the disease [107]. These findings collectively suggest a potential regulatory role for hepatic macrophage apoptosis in the context of liver fibrosis pathogenesis. Specifically, apoptosis may serve to limit the inflammatory accumulation and activation of these cells, thereby promoting disease amelioration.

#### 5.4.3. Necroptosis

Aging is characterized by chronic, low-grade inflammation, which is strongly linked to various age-related diseases, including chronic liver disease and hepatocellular carcinoma. In the liver, this inflammation manifests as increased expression of M1 macrophage markers, pro-inflammatory cytokines (TNFα, IL6, and IL1β), and fibrosis markers. Interestingly, necroptosis, a programmed cell death mechanism, appears to be synchronized with these changes. Studies demonstrate that hepatocytes and hepatic macrophages isolated from aged mice exhibit elevated levels of necroptosis markers and increased expression of pro-inflammatory cytokines compared to their counterparts in young mice. Targeting necroptosis using the inhibitor 7-Cl-O-Nec1 (Nec-1s) offers a promising therapeutic approach. Short-term treatment with Nec-1s in aged mice reduced necroptosis, M1 macrophage markers, fibrosis, cellular senescence, and pro-inflammatory cytokine expression in the liver. This suggests that necroptosis inhibition could be a viable strategy to combat age-related liver damage and potentially other age-related diseases [108]. While the contribution of macrophage necroptosis to hepatic inflammation and fibrosis is attracting increasing research interest, current investigations have primarily focused on NASH [66] and bacterial infections [109]. These findings suggest that macrophages, beyond their established pro-inflammatory and anti-inflammatory roles, additionally contribute to the pathogenesis of inflammatory and fibrotic liver diseases through necroptosis. Notably, necroptosis extends beyond macrophages, affecting other liver cell types during liver fibrogenesis.

#### 5.4.4. Pyroptosis

NLRP3 inflammasome-mediated pyroptosis plays a role in liver fibrosis progression, but the specific intrahepatic cell types involved and their mechanisms remain unclear. Studies suggest that KCs are the primary cell type undergoing pyroptosis in liver fibrosis models. One study reported that S100A8 activates TLR4/NF-κB signaling, upregulates NLRP3, pro-IL-1β, and pro-IL-18, and triggers ROS-mediated activation of NLRP3 inflammasomes in KCs, ultimately leading to pyroptosis and inhibiting liver fibrosis [110]. Following this line of research, another study investigated ursolic acid (UA), a compound with antifibrotic properties. The study found that UA inhibits KC pyroptosis both in vitro and in vivo by suppressing the NOX2/NLRP3 inflammasome pathway, hinting at its potential as a therapeutic target against liver fibrosis [111]. The NADPH oxidase (NOX) family comprises seven members, NOX1–5 and DUOX1–2 [112]. In the liver, NOX2, primarily expressed by KCs, plays a crucial role in defending against microbes and harmful agents [113]. However, a study investigating the potential antifibrotic effects of UA revealed that inhibiting NOX2 in the presence of UA did not significantly reduce KC pyroptosis in CCL4-induced liver fibrosis compared to NOX2 inhibition alone. These findings suggest that UA’s beneficial effect in attenuating liver fibrosis in mice likely extends beyond solely inhibiting the NOX2/NLRP3 inflammasome pathway and KC pyroptosis, although this pathway may still be involved [111]. It is worth noting that KCs exhibit a significantly higher expression of NLRP3 compared to hepatic stellate cells and hepatocytes [114], indicating that KCs serve as the primary site for NLRP3 inflammasome assembly and activation within the liver. However, the impact of NLRP3 inflammasome-mediated pyroptosis of KCs on liver fibrosis remains poorly understood and requires further investigation. Despite the limited data on KC pyroptosis, the potential role of macrophage pyroptosis in various chronic liver diseases, including those with fibrotic outcomes, warrants further exploration. Studies investigating macrophage pyroptosis in liver fibrosis can serve as a valuable foundation for future research, potentially paving the way for novel therapeutic strategies that target this cell death pathway for early intervention.

#### 5.4.5. Ferroptosis

While iron accumulation is common in liver disease, it rarely represents true overload. Instead, it often results from iron that is released from damaged hepatocytes and deposited in macrophages. This phenomenon, coupled with lipid peroxidation, frequently accompanies the onset of ferroptosis. Although less studied in cirrhosis and hepatic fibrosis, macrophage iron deposition has been extensively investigated. Systemic sclerosis, a rheumatic disease with unclear pathogenesis, involves multiorgan inflammation driven by immune dysfunction and eventual organ fibrosis. A recent study employed a mouse model of systemic sclerosis with elevated ACSL4 expression to demonstrate that the acyl-CoA synthetase long-chain family member 4 (ACSL4)-induced ferroptosis of inflammatory macrophages exacerbates fibrosis progression [115]. Separate studies confirmed this link, showing that iron overload (Hfe^−/−^) and Nrf2^−/−^ mice develop increased iron-related necroinflammatory lesions. Specifically, phagocytosis of dead hepatocytes led to macrophage iron deposition in the form of large aggregates. Subsequently, myofibroblasts in the injured area produced abundant collagen fibers, ultimately leading to increased hepatic fibrosis [116]. Chronic hepatitis B virus (HBV) and/or hepatitis C virus (HCV) infection, in the absence of standardized treatment, frequently progresses to liver fibrosis or cirrhosis [117]. Recent studies have revealed a potential link between ferroptosis, a non-apoptotic form of cell death, and immune cell subpopulations. Macrophages, neutrophils, T cells, and B cells exhibit varying degrees of susceptibility to ferroptosis due to changes in gene expression that occur during their maturation process. These findings suggest potential implications for both innate and adaptive immunity [118]. However, the specific role of ferroptosis in the progression of infection-associated liver diseases, such as HBV/HCV infection and fibrosis, remains to be elucidated. Overall, these novel insights into the interplay between ferroptosis and immune function may pave the way for the development of novel antiviral strategies for the treatment of chronic viral hepatitis.

### 5.5. The Effect of Macrophage PCD on Hepatocellular Carcinoma

Despite progress in lowering overall cancer mortality through early screening, the incidence and mortality of HCC are worryingly on the rise [119]. As one of the most prevalent solid tumors globally, HCC ranks as the second leading cause of cancer-related deaths, claiming approximately 830,180 lives in 2020 alone [120]. The 5-year survival rate after liver cancer surgery remains alarmingly low, ranging from only 10 to 18 percent [121]. Therefore, new diagnostic and therapeutic targets must be identified to improve the prognosis of patients with HCC.

#### 5.5.1. Autophagy

Macrophage autophagy plays a complex and multifaceted role in hepatocellular carcinoma development. In the pre-tumorigenic stage, autophagy-deficient KCs are more likely to promote tumor progression, possibly through a mechanism involving activation of the mitochondrial ROS/NF-κB/IL-1α/β pathway [101]. Tumor-associated macrophages (TAMs) play a key role in the tumor microenvironment, and their repolarization towards the M1 phenotype can be beneficial for tumor regression. Interestingly, studies have shown that baicalin, a natural compound, inhibits HCC development by promoting the release of pro-inflammatory cytokines through TAM repolarization to the M1 type [122]. Moreover, the regulatory mechanism behind baicalin-induced autophagy encompasses both lysosomal degradation and TAM repolarization, suggesting a more intricate process than previously understood [122]. These studies provide compelling evidence for the protective role of macrophage autophagy-mediated immune responses in hepatocarcinogenesis. Furthermore, they establish a theoretical foundation for the modulation of macrophage autophagic activity as a potential strategy for both the prevention and treatment of HCC.

#### 5.5.2. Apoptosis

Normally, high levels of AIM circulate in the blood in an inactive state, bound to IgM pentamers. However, under stress, AIM detaches from IgM and acquires disease-repairing properties [123]. The potential link between fructose intake and susceptibility to steatosis-associated hepatocellular carcinoma is an open question. Interestingly, research using a high-fructose diet model showed that mice lacking AIM (AIM^−/−^) had less hepatic steatosis, inflammation, and fibrosis compared to mice fed a high-fat diet. However, despite this apparent protective effect, the study surprisingly found that AIM^−/−^ mice were more susceptible to HCC. Interestingly, AIM itself does not directly induce HCC; instead, it specifically triggers necroptosis (programmed cell death) in AIM-bound cancer cells by activating the complement cascade. Therefore, lower AIM levels during chronic fructose intake might increase HCC susceptibility not by promoting tumor growth but by reducing the elimination of AIM-bound cancer cells [124]. This finding aligns with clinical studies demonstrating that in patients with NASH-HCC, blood levels of activated AIM (free of IgM) are significantly elevated, suggesting its potential as a sensitive diagnostic marker for this specific type of HCC [125]. While research on macrophage apoptosis in HCC has been relatively limited, accumulating evidence suggests its involvement in other liver diseases (NASH, NAFLD, ALD, and liver fibrosis) and even various tumor types. This ongoing exploration of related pathologies serves as a valuable reminder to investigate the regulatory mechanisms of macrophage apoptosis within the context of HCC.

#### 5.5.3. Necroptosis

The immune response of hepatic macrophages to extrahepatic antigens and programmed necroptosis, particularly involving the RIPK3 protein, are intricately linked and play crucial roles in regulating macrophage function, survival, and, ultimately, liver immune homeostasis. As previously discussed, repolarizing tumor-associated macrophages towards the M1 phenotype promotes tumor regression, while the opposite is true for M2 polarization. Interestingly, Wu et al. identified that reduced RIPK3 expression in HCC-associated macrophages promotes M2-type TAM accumulation and polarization, ultimately accelerating tumor progression [126]. This effect is likely due to decreased ROS production and significant inhibition of caspase-1-mediated PPAR cleavage in RIPK3-deficient TAMs. Activated PPAR promotes fatty acid metabolism, including fatty acid oxidation (FAO), which further contributes to M2 polarization in the tumor microenvironment. Necroptosis, a form of regulated cell death, has garnered increasing research interest in the context of tumorigenesis, tumor immunity, and disease progression. Exploring the relationships between necroptosis-related genes and drug sensitivity, HCC prognosis, and particularly necroptosis in immune cells such as macrophages holds promise for the development of novel strategies in HCC risk stratification and treatment optimization.

#### 5.5.4. Pyroptosis

The specific roles of cellular pyroptosis-related genes in tumor progression, immune response, prognosis, and immunotherapy remain incompletely understood. Notably, nonstromal hepatocytes contribute significantly to shaping the tumor microenvironment, with macrophages attracting particular research interest. A study revealed a substantial association between pyroptosis and prognosis in HCC, suggesting that cellular pyroptosis-related genes contribute to the tumor microenvironment’s heterogeneity [127]. Utilizing in vivo macrophage depletion assays and in vitro analyses, researchers demonstrated the critical role of macrophages in mediating anti-tumor effects and identified a strong interdependence between macrophages and NK cells in efficiently eliminating tumor cells. This study further uncovered a novel mechanism of sorafenib treatment: it induces macrophage pyroptosis, prompting the release of NK cells to combat HCC [128]. Hou et al. [129] found that PD-L1 can convert TNFα-induced apoptosis to pyroptosis in cancer cells, ultimately leading to tumor necroptosis. Under hypoxic conditions, p-STAT3 physically interacts with PD-L1, promoting its nuclear translocation and enhancing the transcription of the gasdermin C (GSDMC) gene. GSDMC is specifically cleaved by caspase-8 with TNFα treatment, generating the GSDMC n-terminal structural domain, which forms a pore in the cell membrane and induces pyroptosis. This study introduces the novel concept of GSDMC/caspase-8 mediating an atypical pyroptosis pathway within tumor cells. Pyroptosis is increasingly recognized for its potential anticancer effects across various malignancies. This form of programmed cell death is believed to trigger a robust antitumor immune response, potentially leading to the formation of long-term immunological memory within the body. These characteristics strongly support the designation of pyroptosis as an immunogenic cell death pathway with significant implications for cancer therapy. However, despite these promising findings, the field of pyroptosis-based cancer treatment strategies still awaits comprehensive reviews and summaries of recent advancements.

#### 5.5.5. Ferroptosis

While exogenous ferroptosis inhibitors benefit most liver diseases, HCC is a unique case where inducing ferroptosis holds therapeutic promise. One study employed a transgenic mouse model with a specific knockout of the macrophage cystine/glutamate antiporter xCT (solute carrier family 7 member 11, SLC7A11) gene, demonstrating reduced tumorigenicity and metastasis in the HCC model [101]. Furthermore, xCT (SLC7A11)-mediated ferroptosis in macrophages significantly increased their PD-L1 expression, enhancing their anti-tumor capacity [130]. Deep scRNA-seq analysis of immune cells from HCC patients revealed key insights: tumor-associated macrophages overexpressed apolipoprotein C1(APOC1), which promoted their M2 phenotype. Interestingly, inhibiting APOC1 remodeled the tumor microenvironment by driving TAMs towards the anti-tumor M1 phenotype, potentially via the ferroptosis pathway [131]. Another study identified another contributor to ferroptosis dysregulation in HCC. MiR-142-3p, highly expressed in M1 macrophages of HBV-infected HCC patients, was found to promote ferroptosis in these cells through solute carrier family 3 member 2 (SLC3A2). However, this ferroptosis induction ultimately led to increased production of harmful molecules such as glutathione (GSH), MDA, and Fe^2+^, accelerating HCC development [132]. These findings collectively illuminate the intricate role of ferroptosis in TAM function and HCC progression. Further research focusing on targeting ferroptosis activation in TAMs and regulating their infiltration and functional expression holds promise for achieving precise tumor prevention and improving therapeutic efficacy in HCC.

## 6. Macrophage-Related Biomarkers in Liver Disease

**Figure 4:**
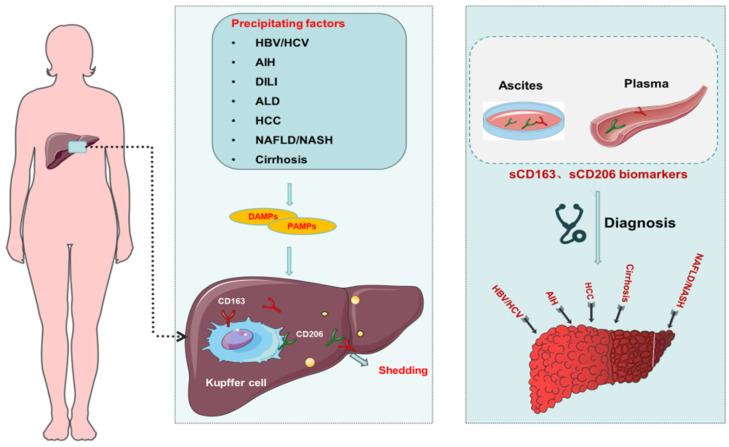
Macrophage Activation Markers, CD163 and CD206, in liver disease. Soluble CD163 and CD206 biomarkers can reveal and quantify the activation of liver macrophages (KCs) as biomarkers of relevant liver disease severity and prognosis. HBV: Hepatitis B virus; HCV: Hepatitis C virus; AIH: Autoimmune hepatitis; DILI: Drug-induced liver injury; ALD: Alcoholic liver disease; HCC: Hepatocellular carcinoma; NAFLD: Non-alcoholic fatty liver disease; NASH: Non-alcoholic steatohepatitis.

Macrophages play a crucial role in the immune response, recognizing both host and foreign molecules through membrane receptors. This recognition can trigger inflammatory responses that recruit and activate other immune cells, impacting various disease processes. In the context of liver disease, specific macrophage-associated biomarkers, CD163 (hemoglobin scavenger receptor) and CD206 (mannose receptor), have gained significant attention [133]. Soluble forms of these receptors, known as sCD163 and sMR/sCD206, have been shown to strongly correlate with the severity and prognosis of various liver diseases, including NAFLD/NASH, viral hepatitis, autoimmune hepatitis, and cirrhosis (Figure 4) [134,135,136,137,138,139,140,141]. Their elevated levels in cirrhotic patients are linked to liver function decline (e.g., MELD and Child–Pugh scores) and portal hypertension severity [142,143]. In addition, sCD163 predicted mortality from variceal hemorrhage and alcoholic hepatitis in cirrhotic patients [142,144]. In patients with spontaneous bacterial peritonitis, another study identified sMR levels in ascites fluid as a marker for peritoneal macrophage activation and inflammation and even predicted 90-day survival [134]. This finding highlights the potential of sMR as a valuable diagnostic tool. Similarly, both sCD163 and sMR in acute-on-chronic liver failure are independently associated with disease severity and prognosis, even improving the accuracy of standard clinical scores [138]. Interestingly, sCD163 and sMR levels were unaffected by the reduction of portal hypertension using a transjugular intrahepatic portosystemic shunt, suggesting that KCs are activated in cirrhotic patients in parallel with portal hypertension. Mechanical reduction of portal hypertension and reduction of signs of endotoxemia did not alleviate this activation. Accordingly, KC activation is a constitutive event that may play a pathogenic role in portal hypertension [145].

## 7. Mechanisms of Macrophage-Targeted Therapy for Liver Diseases and Development of Small Molecule Drugs

Fueled by advancements in molecular biology and tumor immunology, immunotherapy, a revolutionary treatment that empowers the patient’s own immune system, has exploded in oncology and is now being actively explored for a range of non-tumor diseases, such as autoimmune disorders and chronic infections [146]. While current immunotherapy strategies focusing on programmed cell death 1 (PD-1), PD-L1, and cytotoxic T-lymphocyte-associated protein 4 aim to rejuvenate T-lymphocyte function and bolster the acquired immune system, they overlook the critical role of macrophages, the most abundant immune cells, in various disease processes [147]. Macrophages, as major drivers of hepatic inflammation, steatosis, fibrosis, and carcinogenesis, are potential therapeutic targets for the treatment of several liver diseases. Preclinical studies, encompassing phase I, II, and III clinical trials, have yielded promising evidence for macrophage-targeted therapies in achieving remission in various liver diseases, including ALD, NASH, viral hepatitis, and HCC. These findings suggest the potential of macrophage-targeted therapies as viable candidates for personalized treatment approaches (Table 2).

Current research on macrophage-targeted therapies for the liver focuses on five main categories. The first involves regulating macrophage polarization or reprogramming these cells to a desired state. Additionally, nanoparticles are being explored as targeted delivery vehicles due to the strong scavenging ability of macrophages. These nanoparticles can be modified to bind specifically to macrophages, reducing unwanted uptake by other cells. This targeted approach allows for the release of drugs at the desired site, alters immune cell response, and extends the circulation time of the nanoparticles, ultimately reducing their toxicity. For instance, the spleen tyrosine kinase inhibitor R406, encapsulated in modified nanoparticles, demonstrates efficient drug delivery and attenuates choline-deficient diet-induced NASH by inhibiting immune cell infiltration and macrophage activation [148]. Secondly, researchers are investigating methods to inhibit monocyte recruitment to the liver. MoMFs are recruited by KCs to amplify and perpetuate hepatic inflammation. This recruitment process is driven by interactions between chemokine receptors, such as CCL2 and CCR2. Therefore, strategies to reduce MoMF infiltration involve targeting or interfering with this chemokine signaling pathway [149]. Thirdly, therapies aim to inhibit the activation and injury response of KCs. When these resident macrophages recognize liver injury, they initiate a cascade of inflammatory responses. Studies using a co-culture system of mouse primary hepatocytes and KCs have shown that pyroptosis-induced injury signals from hepatocytes activate KCs. This activation creates a feedback loop through pro-inflammatory signaling that amplifies NLRP3-dependent hepatocyte pyroptosis, ultimately leading to more severe hepatic inflammation [150]. Fourthly, some therapies focus on depleting or eliminating TAMs in HCC. This can be achieved through various means, including directly eliminating TAMs from tumors, blocking the recruitment of MoMFs, or reprogramming TAMs to an anti-tumor phenotype. Liposomes, artificially created vesicles, can be used to deliver clodronate, a drug that induces macrophage apoptosis after being taken up by these cells. Clodronate-encapsulated liposomes have been shown to partially eliminate TAMs and slow tumor growth, particularly by reducing the M2-type TAM population. This suggests that eliminating a majority of TAMs may trigger phenotypic changes in the remaining ones [151]. Finally, therapies can target the downstream effects of TAM products. TAMs are a significant source of the paracrine signaling molecule IL-6 during HCC progression. Tocilizumab, an anti-IL-6 receptor antibody used for rheumatoid arthritis, has been shown to block IL-6 signaling and inhibit TAM-stimulated tumor stem cell activity in both in vitro and in vivo models [152]. While these strategies have shown promise in animal models, translating macrophage-targeted therapies to humans remains challenging due to the existence of unique macrophage subpopulations and the variable nature of disease progression. This field urgently needs a breakthrough to accelerate research and development efforts to unlock the full potential of macrophage-targeted therapies for a wider range of liver diseases.

**Table 2:** Overview of clinical studies of small molecule drugs targeting macrophage modulation.

Target	Mechanism	Drug	Clinical Trial/Phase	Clinical Trial Number or Reference
**ALD**				
antibiotic	Attack intestinal bacteria and inhibit macrophage activation	Vancomycin, gentamicin, meropenem	I	NCT03157388 [153]
IL-1β antagonist	Inhibition of inflammasome activation in KCs	IL-1Ra	II	NCT01809132 [154]
**NASH**				
FXR agonists	Increased cholesterol transport in macrophages	Obeticholic acid	III	NCT02548351 [155]
CCR2/CCR5 antagonist	Inhibit monocyte recruitment	Cenicriviroc	II	NCT02217475 [156]
Galectin-3 antagonist	Inhibition of inflammatory macrophage function	GR-MD-02	II	NCT02462967 [157]
PPARα/δ agonist	Promote differentiation of macrophages into anti-inflammatory subgroups	Elafibranor	III	NCT02704403 [158]
**Viral hepatitis**				
GM-CSF	GM-CSF promotes macrophage differentiation	Y peginterferon alpha-2b plus GM-CSF	II	NCT02332473 [159]
**HCC**				
PD-1/PD-L1	Regulation of immune checkpoints in macrophages	CA-170	II	NCT04343859 [160]
CSF1R	Multi-target inhibitor that suppresses angiogenesis-related kinases and decreases macrophage differentiation.	Chiauranib	I	NCT03245190
CCR2/5	CCR2/CCR5 antagonist (inhibits monocyte/macrophage infiltration)	Nivolumab plus CCR2/5 inhibitor	II	NCT04123379

FXR: Farnesoid X receptor; IL-1β: Interleukin-1β; IL-1Ra: Interleukin-1 receptor antagonist; CCR5: Chemokine receptor 5; CCR2: Chemokine receptor 2; GR-MD-02: Belapectin; PPARα/δ: Peroxisome proliferator-activator receptors α/δ: PD-1: Programmed cell death 1; PD-L1: Programmed cell death ligand 1; GM-CSF: granulocyte-macrophage colony stimulating factor; CSF1R: colony stimulating factor 1 receptor.

## 8. Conclusions and Prospects

Understanding how programmed cell death manifests differently in different cells and environments is critical to shedding further light on the complex immune mechanisms of organisms. This review delves into macrophage PCD, highlighting the double-edged sword of its nature: protecting organs from chronic inflammation and pathogen invasion while potentially accelerating tissue damage. However, the regulatory mechanisms linking macrophage PCD to its polarization state remain elusive. Importantly, it should be borne in mind that various cell death modes are not isolated pathways but engage in complex “crosstalk”, influencing each other. Despite its immense potential, PCD remains underutilized in clinical practice. The future holds great promise in leveraging 3D cultures, where researchers can cultivate human organoids (organoids) in vitro for experimentation. This approach can accelerate the translation of cell death biology into practical clinical applications, potentially revolutionizing diagnosis, monitoring, and treatment strategies for liver and other immune-inflammatory diseases. By unlocking the power of PCD, we may be able to block or delay disease progression at an early stage and significantly improve cure rates. Due to the heterogeneity of macrophages in liver diseases, future basic and clinical studies may be conducted in the following areas: ① the significance and functions of different macrophage phenotypes in the course of liver diseases; ② whether the PCD of macrophages can be selectively regulated to address the development of liver fibrosis/hepatocellular carcinoma; ③ the mechanism of in vivo macrophage therapy without affecting the functions of other cells; ④ how to better utilize molecularly targeted drugs to inhibit the negative effects of pro-inflammatory and polarized liver macrophages or promote their positive anti-inflammatory and anti-fibrotic effects.

## Figures and Tables

**Table 1:** Different PCD modes of cells.

Cell Death Type	Death Pathway/Mechanism	Key Factor	Feature	Inducer
Autophagy	1. mTOR pathway 2. MAPK pathway 3. Beclin 1 pathway 4. PI3K/Akt pathway 5. ROS pathway 6. NF-κB pathway	LC3 Beclin-1	Autophagosome formation	1. Hungry 2. Oxygen deficit 3. Bacterial or viral infections
Apoptosis	1. Extrinsic pathway induced by TNF or TRAIL 2. Intrinsic (mitochondrial) pathway mediated by pro-apoptotic Bcl-2 family proteins 3. Endoplasmic reticulum stress-induced pathway	Caspase3 Caspase8 DISC Caspase 9 MOMP	1. Cell shrinkage, but cell membrane integrity 2. Chromatin condensation 3. DNA fragmentation 4. Apoptotic body formation	1. Bacterial infections 2. Hypoxia 3. Chemicals
Necroptosis	1. TNFR1-RIPK1 pathway 2. RIPK3-MLKL pathway	RIPK1 RIPK3 MLKL	1. Cell membrane rupture 2. Cell content extravasation 3. Inflammatory response	1. Bacterial toxin 2. Oxidative stress 3. Cell trauma 4. Pathogen 5. Nutritional deficiency 6. ATP depletion 7. Mitochondrial permeability transition
Pyroptosis	1. Caspase1-dependent pathway 2. Caspase11/4/5-independent pathway	GSDMD	1. Cell swelling 2. Cell membrane rupture 3. Release of IL-1β 4. ASC-mediated inflammasome Formation 5. Create pores in the cell membrane	1. Cytosolic dsDNA 2. Anthrax lethal toxin 3. Membrane damage 4. Toxoplasma 5. Chemotherapy drugs
Ferroptosis	Excess Fe^3+^ in cell is reduced to Fe^2+^ to form hydroxyl radicals, leading to accumulation of lipid peroxides, resulting in increased ROS	GPX4	1. Smaller outer membrane of mitochondria ruptures 2. MPK mediated Beclin-1 phosphorylation	1. GSH depletion 2. Inactivation of GPX4

TNF: Tumor necrosis factor; TRAIL: Tumor necrosis factor-related apoptosis-inducing ligand; Bcl-2:B-cell lymphoma 2; DISC: Death-inducing signaling complex; TNFR1: The Tumor Necrosis Factor Receptor-1; RIPK1: Receptor interacting protein kinases 1; RIPK3: Receptor interacting protein kinases 3; MLKL: Mixed lineage kinase domain protein; mTOR: Mechanistic target of rapamycin; MAPK: Mitogen-activated protein kinase; PI3K: Phosphoinositide 3-kinase; AKT: Protein kinase B; ROS: Reactive oxygen species; GSDMD: Dasdermin D; ASC: Apoptosis-associated speck-like protein; NF-κB; Nuclear factor kappa-B; GPX4:Glutathione peroxidase 4; GSH: Glutathione; MPK: Mitogen-activated protein kinase.

## Abbreviations

TNF: Tumor necrosis factor; TRAIL: Tumor necrosis factor-related apoptosis-inducing ligand; Bcl-2:B-cell lymphoma 2; DISC: Death-inducing signaling complex; TNFR1: The Tumor Necrosis Factor Receptor-1; RIPK1: Receptor interacting protein kinases 1; RIPK3: Receptor interacting protein kinases 3; MLKL: Mixed lineage kinase domain protein; mTOR: Mechanistic target of rapamycin; MAPK: Mitogen-activated protein kinase; PI3K: Phosphoinositide 3-kinase; AKT: Protein kinase B; ROS: Reactive oxygen species; GSDMD: Gasdermin D; ASC: Apoptosis-associated speck-like protein; NF-κB; Nuclear factor kappa-B; GPX4:Glutathione peroxidase 4; GSH: Glutathione; MPK: Mitogen-activated protein kinase; NO: Nitric oxide; IL: Interleukin; Arg-1: Arginase-1; TG: Triglyceride; HDL: High density lipoprotein; TAMs: Tumor-associated macrophages; HIF-1a: Hypoxia inducible factor-1a; FXR: Farnesoid X receptor; IL-1Ra: Interleukin-1 receptor antagonist; CCR5: Chemokine receptor 5; CCR2: Chemokine receptor 2; GR-MD-02: Belapectin; Peroxisome proliferator-activator receptors α/δ: PPARα/δ; PD-1: Programmed cell death 1; PD-L1: Programmed cell death ligand 1; LPS: Lipopolysaccharide; MHC-II: Histocompatibility complex class II; TGF-β: Transforming growth factor-b; TLR4: Toll-like receptor 4; Class III PI3K:Class III phosphoinositide 3-kinase; mTOR: Mechanistic target of rapamycin; LC3: Microtubule-associated protein light chain 3; ATG5: Autophagy protein 5; Bcl-2: B-cell lymphoma 2; TRADD: TNFR1-associated death domain protein; PIPK1: Phosphatidylinositol phosphate kinase 1; PIPK3: Phosphatidylinositol phosphate kinase 3; SLC3A2:Solute carrier family 3 member 2; SLC7A11:Solute carrier family 7 member 11; CoQ: Coenzyme Q; FSP1:Ferroptosis suppressor protein 1; ERK: Extracellular signal-regulated kinases; PIPK1: Phosphatidylinositol phosphate kinase 1; PIPK3: Phosphatidylinositol phosphate kinase 3; AMPK: AMP-activated Protein Kinase; ULK1:UNC-51-like kinase 1; BCL2:B-cell lymphoma 2; TRAF2:TNF receptor associated factor 2; TRAF5: TNF receptor associated factor 2; HBV: Hepatitis B virus; HCV: Hepatitis C virus; AIH: Autoimmune hepatitis; DILI: Drug-induced liver injury; ALD: Alcoholic liver disease; HCC: Hepatocellular carcinoma; NAFLD: Non-alcoholic fatty liver disease; NASH: Non-alcoholic steatohepatitis.
